# Mapping the evidence on health equity considerations in economic evaluations of health interventions: a scoping review protocol

**DOI:** 10.1186/s13643-019-1257-4

**Published:** 2020-01-08

**Authors:** Hafizah Besar Sa’aid, Sharon Mathew, Marina Richardson, Joanna M. Bielecki, Beate Sander

**Affiliations:** 1 Toronto Health Economics and Technology Assessment (THETA) Collaborative, University Health Network, Toronto, Ontario Canada; 2 Faculty of Business and Management, Universiti Teknologi MARA (UiTM), Sungai Petani, Kedah Malaysia; 3 Institute of Health Policy, Management and Evaluation, University of Toronto, Toronto, Ontario Canada; 4 Public Health Ontario, Toronto, Ontario Canada; 5 ICES, Toronto, Ontario Canada

**Keywords:** Health equity, Economic evaluations, Health interventions, Scoping review, Decision-making

## Abstract

**Background:**

Equity in health has become an important policy agenda around the world, prompting health economists to advance methods to enable the inclusion of equity in economic evaluations. Among the methods that have been proposed to explicitly include equity are the weighting analysis, equity impact analysis, and equity trade-off analysis. This is a new development and a comprehensive overview of trends and concepts of health equity in economic evaluations is lacking. Thus, our objective is to map the current state of the literature with respect to how health equity is considered in economic evaluations of health interventions reported in the academic and gray literature.

**Methods:**

We will conduct a scoping review to identify and map evidence on how health equity is considered in economic evaluations of health interventions. We will search relevant electronic, gray literature and key journals. We developed a search strategy using text words and Medical Subject Headings terms related to health equity and economic evaluations of health interventions. Articles retrieved will be uploaded to reference manager software for screening and data extraction. Two reviewers will independently screen the articles based on their titles and abstracts for inclusion, and then will independently screen a full text to ascertain final inclusion. A simple numerical count will be used to quantify the data and a content analysis will be conducted to present the narrative; that is, a thematic summary of the data collected.

**Discussion:**

The results of this scoping review will provide a comprehensive overview of the current evidence on how health equity is considered in economic evaluations of health interventions and its research gaps. It will also provide key information to decision-makers and policy-makers to understand ways to include health equity into the prioritization of health interventions when aiming for a more equitable distribution of health resources.

**Systematic review registration:**

This protocol was registered with Open Science Framework (OSF) Registry on August 14, 2019 (https://osf.io/9my2z/registrations).

## Background

Maximization of health, reduction of inequities in health, and financial protection against the costs of ill health are three central tenets of priority setting for health interventions [1]. However, the present priority setting methods often focus on health maximization and do not adequately capture the complexities of equity and financial protection [1]. The goal of achieving equity in health has become central to the post-2015 United Nations’ (UN) Development Agenda [2] and the UN’s 2030 Agenda for Sustainable Development [3]. The influence of these mandates is evident in advances in the methods of economic evaluation of health interventions, where health equity has been introduced as an important consideration [4]. However, a comprehensive study that maps the trends and concepts of health equity in health economic evaluations of health interventions has yet to be conducted. In response to this perceived gap, our objective is to map the existing knowledge on how health equity is considered in economic evaluations of health interventions as reported in the academic and gray literature.

The objective of this protocol is to provide the rationale, scope, and methods for the conduct of the scoping review. This protocol will start with the background information: the definition of health equity, why equity is important to be considered in prioritizing the health interventions, and how equity is an important consideration in health economic evaluations.

### Health equity: definition and importance

Equity is grounded in principles of human rights and distributive justice [5]. The concept of equity is intrinsically normative (value-based), and “…a matter of morality, fairness and justice” [6]. Consequently, health equity refers to the absence of unjust or unfair health disparities, i.e., preventable or remediable differences in one or more determinants of health across populations or population groups [7, 8]. The WHO elaborates further that “Health inequities involve more than inequality with respect to health determinants, access to resources needed to improve and maintain health and health outcomes”, and thus health equity refers to the fair distribution of health in the population.

Health equity can be divided into horizontal and vertical equity. Horizontal equity means equal access to health for all population groups (i.e., people of equal needs should be treated the same), whereas vertical equity means inclusion of all needed services (i.e., people with greater clinical needs should have more intervention than those with fewer needs) [9, 10]. Equity is an important topic on the world policy agenda because of disparities in health among population groups (between rich and poor, urban and rural, employed and unemployed) and countries (between low-, middle- and high-income countries).

Equity has been at the forefront of the WHO’s agenda since 1946. In 1978, participant countries at the Alma-Ata WHO Conference endorsed the concept of health equity, which resulted in launching the “Health for All (HFA)” campaign [11]. Since then, equity in health has become an important part of the policy agenda for all countries. In 2000, eight Millennium Development Goals (MDGs) were established by the United Nations (UN) to address health inequity by 2015 [3]. As continuity, the UN launched the 17 Sustainable Development Goals (SDGs) that need to be achieved by 2030. In these goals, health equity has become the soul of the agenda [3].

Despite the declared commitment to health equity, most health systems around the world (from macro to micro) are still routinely making decisions on choosing new health interventions based on economic evaluations and maximizing health outcomes, often measured in quality-adjusted life-years (QALYs) [12, 13]. Hence, decision-makers intrinsically balance quality of care and financial outcome parameters [14], but rarely make decisions based on the trade-offs between the economic and equity distributions of the health outcomes [4].

### Health equity as a part of health economic evaluations

Nevertheless, the rise of health equity on the policy agenda around the world has triggered health economists to develop methods to explicitly include equity in health economic evaluations of new health interventions in order to help policy- and decision-makers make informed decisions with an equity focus. Among the methods that have been proposed to quantify health inequity are rate ratios, population attributable risks, slope and relative indices of inequality, and the concentration curve and index [15]. Other methods include the equity weighting analysis [16], equity impact analysis, and equity trade-off analysis [4]. Still, until today, decision-making bodies have yet to adopt these methods to consider equity in their decisions. If decision-makers consider equity, it is usually informal, ad hoc, simplistic and lacking transparency [16]. Evidence on how to improve health equity in the most efficient way is vital [17].

Since the 1978 Declaration of Alma-Ata, citations in MEDLINE that include the term “equity” increased by 216% in 2015 compared to 1980 [18]. Given the increase in publications, comprehensive reviews and analyses on equity consideration in economic evaluations have also been conducted, though most reviews have focused on specific methodologies of how to integrate equity concerns into economic evaluations [19–21]. Other reviews have been based on specific interventions or concepts, such as vaccines, operation research, and universal health coverage [9, 18, 22]. However, no review has comprehensively mapped the existing knowledge to date in a scoping review.

Scoping reviews follow a rigorous and transparent process in identifying relevant literature to map the broad knowledge instead of summing up the best available research on a specific question [23]. In our scoping review, we will map the current state of trends and concepts of how health equity is considered in health economic evaluations both empirically and methodologically, and synthesize the literature to identify any knowledge gaps.

## Methods

### Study design

Our protocol is guided by the scoping review framework proposed by Arksey and O’Malley, which was refined further by Levac et al. and the Joanna Briggs Institute [24–26]. The overview of the stages for the scoping review process as proposed by Arksey and O’Malley is depicted in Table 1 [24, 25]. The populated PRISMA-P checklist [27] for this study protocol is attached as an additional file (see Additional file 1).

**Table 1:** The original scoping review framework proposed by Arksey and O’Malley [24, 25]

Stage		Description
1.	Identifying the research question	• As a guide to build a research strategy. • Should be wide enough to generate a breadth of coverage.
2.	Identifying relevant studies	• Via different sources: electronic databases, reference lists, hand-searching of key journals, existing networks, relevant organizations, and conferences. • Make decision about the coverage of the review in terms of time span and language (consider time and budget constraints).
3.	Study selection	• The criteria for the inclusion/exclusion criteria are device post hoc based on increasing familiarity with the subject matter through reading the studies. • Use an iterative team approach to select the studies and extract the data.
4.	Charting the data	• The work involves “charting” key items of the information obtained from the primary research reports being reviewed. • A data-charting form is developed and used to extract data from each study.
5.	Collating, summarizing, and reporting the results	• An analytic framework or thematic construction is used to provide an overview of the breadth of the literature but not a synthesis. • A numerical analysis of the extent and nature of the studies using tables and charts is presented. A thematic analysis is then presented. • Clarity and consistency are required when reporting results.
6.	Consultation exercise (optional)	• Provides opportunities for consumer and stakeholder involvement to suggest additional references and provide insights beyond those in the literature.

The original framework proposed by Arksey and O’Malley [24] comprised six stages, including an optional consultation with key stakeholders to identify additional references and gather feedback on the findings of the scoping review. While a consultation with stakeholders would be a valuable exercise, we will not include that optional stage in this scoping review because of budget and time constraints. Our protocol was registered with Open Science Framework (OSF) Registry on August 14, 2019 (https://osf.io/9my2z/registrations).

### Stage 1: identifying the research question

The purpose of this scoping review is to map the current state of the literature on the trends and concepts of how health equity is considered in health economic evaluations of health interventions, and synthesize the literature to identify knowledge gaps. We will include methods papers and empirical studies that consider health equity in full economic evaluations such as cost-utility analysis, cost-effectiveness analysis, cost-benefit analysis, and cost-minimization analysis.

To identify our research questions, we conducted an exploratory review of the literature on health equity in health economic evaluations of health interventions. Based on this exploratory review of the literature, our overarching research question is “What is known in the literature about how health equity is considered in economic evaluations of health interventions?” and our specific research questions are:

1. How is health equity incorporated in economic evaluations of health interventions?
2. For which types of health interventions is health equity included as part of the economic evaluations?
3. How is health equity conceptualized in the economic evaluations of health interventions?
4. Which types of economic evaluations consider health equity in the assessment of health interventions?
5. Which methods are currently used to incorporate health equity in economic evaluations of health interventions?
6. In which settings is health equity considered in economic evaluations of health interventions?
7. Which new methods are proposed for incorporating health equity in economic evaluations of health interventions?
8. Did the conceptualization of health equity considered in economic evaluations of health interventions change over time? If so, how?
9. What are the knowledge gaps in incorporating health equity in economic evaluations of health interventions?

### Stage 2: identifying relevant studies

Based on the Joanna Briggs Institute Reviewers’ Manual 2015 (Methodology for JBI Scoping Reviews) [26], the aim for the second stage of scoping reviews is to identify the criteria that will be used to select the studies, i.e., inclusion criteria to guide the search and help filter for relevant sources.

We searched the following eight databases for relevant articles: MEDLINE Ovid, EMBASE Ovid, EconLit EBSCO, Scopus Elsevier, CINAHL EBSCO, Cochrane Database of Systematic Reviews (CDSR), Proquest (Thesis and Dissertations), and Proquest University of Toronto (Thesis and Dissertations) from the date of inception to May 2019. We have not limited our analysis to economic evaluations published in journals or publications that have undergone formal review as study quality is not assessed for scoping reviews [24, 26]. We also searched for gray literature by following the methods outlined in “Grey Matters: a practical tool for searching health-related grey literature” [28].

The search strategy was designed by a medical research librarian (JB) and it consists of both text words and Medical Subject Headings (MeSH) terms related to economic evaluations and equity. We conducted the search in English. We used published and validated filters to search the following conceptual areas:

1. Health equity (equity, inequity, social determinants of health, health disparity) [29].
2. Economic evaluation (decision model, cost-effectiveness, cost-utility, cost-benefit, pharmaco-economics, cost minimization, cost-efficiency analysis) [30].

Further, we will screen the articles retrieved based on their titles and abstracts, and then a full-text. For full-text screening, we will make every effort to screen all included full-text papers in any language. We will search the reference lists of included full-text studies and hand search key journals to identify any other relevant publications. The articles retrieved will be uploaded to DistillerSR, a reference manager software, which will be also used for data management for further screening and data extraction.

Based on the initial exploratory search and discussions among the review team members and the medical research librarian (JB), our inclusion criteria are:

- Source of information: Publications that are accessible publicly and through University of Toronto and University Health Network libraries
- Time frame: From inception to May 2019
- Language: Abstracts in English language; full-text in any languages
- Research location: Worldwide
- Study population: Human
- Types of interventions: All types of health interventions (medical devices, drugs, procedures, treatments, public health interventions, etc.)
- Type of economic evaluations: Full economic evaluations (cost-effectiveness analysis, cost-utility analysis, cost-minimization analysis, and cost-benefit analysis)
- Type of methods incorporating equity: Any methods of incorporating health equity in economic evaluations (rate ratios, equity weighting analysis, equity impact analysis, equity trade-off analysis, etc.)
- Type of studies: Both empirical (applied) and methods (concept) studies
- Types of articles: All types of study designs, applied studies, concept discussion papers, reviews, meta-analyses, books, theses and dissertations, gray literature, and general articles (including commentaries or editorial articles)

The search strategy for MEDLINE Ovid, together with CLHTA, CLEED, and CDSR, and gray literature are included as additional files (see Additional file 2 and Additional file 3).

### Stage 3: study selection

Selection will be performed based on the inclusion criteria pre-specified in stage 2 of the review protocol and will be conducted in the following two-step process.

Step 1: We will import and de-duplicate the search results from all sources in DistillerSR. Two reviewers will conduct title and abstract screening. All types of articles on health equity considerations in economic evaluations of health interventions as per inclusion criteria in stage 2 will be included. The reviewers will screen each article based on the following question: “Does the article discuss on how health equity is considered in economic evaluations of health intervention?”

To increase consistency among reviewers, all reviewers will first screen the same 100 titles and abstracts and discuss conflicts until a consensus is reached. If consensus cannot be reached, conflicts will be brought to the larger team for further discussion. The reviewers will then screen the remaining citations independently (two reviewers per article) and remove irrelevant studies. For those fulfilling the eligibility criteria, the full-text articles will be retrieved.

Step 2: The full-text articles will be screened independently by the reviewers (two reviewers per article) against the inclusion/exclusion criteria and the rationale for exclusion will be documented. Any eligibility discrepancies will be discussed between reviewers until consensus is reached or will be brought to the larger team for discussion if consensus cannot be reached. We will exclude book reviews, letters, and conference abstracts. Studies remaining at the end of Stage 2 will be included in the review and a rationale for inclusion will be provided.

### Stage 4: charting the data

In stage 4, we will develop a standardized data extraction template to allow the reviewers to categorize or “chart” the data. There are six main categories and 26 subcategories that will be used to assess the full review of the articles that are retrieved after fulfilling the eligible inclusion criteria. We will extract the standard bibliographical information (such as authors, title, journal, and year of publication), study details (such as study designs, data type, settings, and outcomes——if applicable), and what, why, and how health equity was considered (Table 2).

**Table 2:** Data extraction charting template

Main category/subcategories	Description
Article details	
Author(s)	Who are the author(s)?
Title	Full title of the article
Objective(s)	Describe the stated objective(s)
Year of publication	Article year
Article type	What is the type of article?
Language	In which language is the article written?
Country	In which country was the study conducted?
Study details (if applicable)	
Type of study	Empirical or methods?
Study design	Type of methodology
Data type	Primary or secondary data
Study population	Which population was studied?
Health intervention	
Name	Name of the intervention(s)
Type of intervention	What was the type of intervention? (e.g., vaccine, drugs, medical devices, treatment, procedure, and public health)
Settings	Where did the intervention/study take place?
Health equity	
Reasons	Why did the study assess health equity?
Type of health equity	Which type of health equity was considered? (e.g., horizontal and vertical equity, equal utilization, distribution according to need, equal access, and equal health outcomes)
Social determinants of health (if applicable)	Which social determinants of health were addressed?
Other equity measures	Which other equity measures were addressed? (e.g., demographic factors and financial risk protection (FRP))
Evaluation method	How was health equity assessed? (e.g., rate ratios, population attributable risks, equity weighting analysis, equity impact analysis, and equity trade-off analysis)
Tool used	Which tool was used or developed to assess health equity? (e.g., framework and guidelines)
Health economic evaluations	
Type of evaluations	Which type of economic evaluation was conducted?
Evaluation method	How was the economic evaluation conducted? (e.g., administrative data, and clinical trials)
Tool used	Which tool was used or developed? (e.g., framework and guidelines)
Incorporating health equity	
Method development	How does health equity incorporated in the economic evaluations of health interventions?
New method	What are the new methods proposed?
Method practicality	Are there any empirical studies that used the method proposed?
Recommendations	What recommendations did the authors make based on the study results and their experiences?

The reviewers will pilot test the template on a sample of the included studies (i.e., 10% of the included studies) to make sure that the charting is consistently applied. If necessary, we will refine and revise the template accordingly through the course of the review process. If questions arise during the pilot test, the team will resolve the issues through discussion. The same reviewers will extract and chart the data from each included article independently (two reviewers per article) based on the data extraction template. We will make every effort to identify native speakers which can extract the data of all included studies published in languages other than English.

### Stage 5: collating, summarizing, and reporting the results

The data analysis will provide an overview of the breadth of research which in turn will inform of the current status and the way forward in considering health equity in economic evaluations of health interventions. We will follow the PRISMA-ScR checklist [31] and Arksey and O’Malley’s [24] methods of reporting to provide a descriptive numerical analysis of the topic, including the extent, characteristics, and distribution of the included studies. The PRISMA flow diagram for the scoping review process, as suggested by the Joanna Briggs Institute [26] in their scoping review manual, will also be observed. We will first provide a narrative; that is, a thematic summary of the data collected. We will then discuss the results based on the research questions and purpose of the study. We will also highlight areas that are under-researched and may need further investigation.

We will report trends over time for methods and empirical studies of health equity considerations in economic evaluations of health interventions. We will explore gaps between the conceptual thinking about health equity and the application of these methods. We will present the results in aggregate and visual forms (e.g., tables, charts, evidence and concept maps, and bubble plots) as appropriate.

## Discussion and dissemination

We anticipate that the results of this scoping review will provide a comprehensive overview of the evidence on health equity considerations in economic evaluations of health interventions and identify research gaps. The review will also provide key information to decision-makers and policy-makers at the macro and micro levels to understand ways of including health equity into the prioritization of health interventions when aiming for a more equitable distribution of health resources. We will disseminate the findings of this scoping review through publication in a scientific journal and presentation at relevant conferences. This protocol reports a comprehensive and transparent methodology that will inform a rigorous review of equity considerations in economic evaluations of health interventions and lay a foundation for future work in the field.

## Supplementary information

**Additional file 1.** PRISMA-P Checklist. (docx 37kb)

**Additional file 2.** Search strategy for Ovid MEDLINE, CLHTA + CLEED + CDSR. (docx 21kb)

**Additional file 3.** Search strategy for grey literature. (docx 16kb)

## Data Availability

Data sharing is not applicable to this article as no datasets were generated or analyzed during the current study.

## Abbreviations

HFA: Health for All
MDGs: Millennium Development Goals
MeSH: Medical Subject Headings
PRISMA: Preferred Reporting Items for Systematic Reviews and Meta-Analyses
PRISMA-P: Preferred Reporting Items for Systematic Reviews and Meta-Analyses – Protocol
PRISMA-ScR: Preferred Reporting Items for Systematic Reviews and Meta-Analyses – Scoping Review
QALYs: Quality-adjusted life-years
SDGs: Sustainable Development Goals
UN: United Nations
WHO: World Health Organization
