# Supplementary material for: Mapping the evidence on health equity considerations in economic evaluations of health interventions: a scoping review protocol
Source: Syst Rev. 2020 Jan 8;9:6. doi: 10.1186/s13643-019-1257-4 (PMC6950907; doi:10.1186/s13643-019-1257-4)
Supplement: Supplementary file 2 — Additional file 2. Search strategy for Ovid MEDLINE, CLHTA + CLEED + CDSR. (docx 21kb) [file 13643_2019_1257_MOESM2_ESM.docx]

**ADDITIONAL FILE 2**

**SEARCH STRATEGY FOR Ovid MEDLINE, CLHTA + CLEED + CDSR**

**Title: Mapping the evidence on health equity considerations in economic evaluations of health interventions: a scoping review protocol.**

**Limits:** humans, publication types (excluded: editorials, letters, and historical articles)

**Filters:** Published, validated filters for equity based studies and Economic Evaluations (Note: original published filters for Medline and Embase have been translated to CLHTA, CLEED, CDSR (Cochrane), CINAHL and EconLit):

Prady SL, Uphoff EP, Power M, Golder S.[**Development and validation of a search filter to identify equity-focused studies: reducing the number needed to screen**](https://www.ncbi.nlm.nih.gov/pubmed/30314471). BMC Med Res Methodol. 2018;18(1):106.

Glanville J, Fleetwood K, Yellowlees A, Kaunelis D, Mensinkai S**.** [**Development and Testing of Search Filters to Identify Economic Evaluations in MEDLINE and EMBASE**](https://www.cadth.ca/media/pdf/H0490_Search_Filters_for_Economic_Evaluations_mg_e.pdf). Ottawa: Canadian Agency for Drugs and Technologies in Health; 2009

**MEDLINE + CLHTA + CLEED + CDSR (Cochrane) SEARCH**

**Databases searched:**

Ovid MEDLINE(R) ALL 1946 to May 03, 2019,

Ovid Cochrane Database of Systematic Reviews (CDSR), 2005 to May 07, 2019,

Ovid Health Technology Assessment (CLHTA), 4th Quarter 2016,

Ovid NHS Economic Evaluation Database (CLEED), 1st Quarter 2016.

**Search Strategy:**

| **#** | **Searches** | **Results** |
| --- | --- | --- |
| 1 | Residence Characteristics/ or Environment design/ or exp Marital status/ or (neighbo?rhood* or residential environment* or rural* or inner?city or housing instability or housing insecurity or housing strain or housing security or mortgage problems or foreclosure or eviction* or housing loss or home repossession* or home ownership or (repossess* adj3 hous*) or (repossess* adj3 propert*) or mortgage delinquency or mortgage arrears or mortgage debt* or overcrowding or (living adj1 (outside or inside or near* or adjacent)) or (household adj2 size) or (marital status or marriage status) or (widow* or cohabit* or divorce* or single parent* or live* alone)).mp. | 274855 |
| 2 | Cultural Deprivation/ or Acculturation/ or Culture/ or Cross-Cultural Comparison/ or Cultural Characteristics/ or Cultural Diversity/ or Language/ or "Transients and Migrants"/ or exp "Emigrants and Immigrants"/ or Minority groups/ or Minority health/ or Prejudice/ or Racism/ or Xenophobia/ or Social Discrimination/ or exp Race Relations/ or exp Ethnic Groups/ or exp Continental Population Groups/ or Refugees/ or (minorit* or migration background or racial or racism or ethnology or race or ethnic* or non?English or language other than or latino* or latina* or hispanic* or whites or caucasian* or non?white or Torres Strait Islander or aboriginal or native american or inuit or eskimo or first nation* or indigenous or english as a second language or foreign language).mp. | 709096 |
| 3 | Occupations/ or Unemployment/ or (occupations or unemployment).mp. | 55398 |
| 4 | exp Gender Identity/ or Women's Health/ or Sex factors/ or (gender differences or (sex disparit* or sex difference?) or gender identity or sex role or wom#n* role? or m#n* role? or gender* role? or servicewomen).mp. | 339435 |
| 5 | exp Educational status/ or Education/ or (schooling or educational status or (education* adj2 level?) or ((higher or better or worse or less) adj educated) or ((higher or better or worse or less) adj level? of education)).mp. | 113858 |
| 6 | Religion/ or religi*.mp. | 59803 |
| 7 | Social determinants of Health/ or Psychosocial Deprivation/ or Sociological Factors/ or Working Poor/ or Hierarchy, Social/ or (disparit* or inequalit* or inequit* or equity or deprivation or gini or concentration index).mp. or Socioeconomic Factors/ or Social Welfare/ or exp Social Class/ or exp Poverty/ or Income/ or (Social class* or social determinants or social status or social position or social background or social circumstance* or socio-economic or socioeconomic or sociodemographic or socio-demographic or SES or disadvantaged or impoverished or poverty or economic level or assets index or income*).mp. | 578135 |
| 8 | Social Stigma/ or social capital/ or Social Control, Informal/ or exp Social Support/ or exp Social Environment/ or Trust/ or Social conditions/ or Social isolation/ or Social marginalization/ or Anomie/ or social participation/ or (social exclusion or (social adj (capital or cohes* or organis* or organiz*)) or (community adj3 (cohes* or participa*)) or ((neighbourhood or neighborhood) adj cohes*) or social relationships or social network* or collective efficacy or civil society or informal social control or neighbo*rhood disorder or social disorgani?ation or anomie or social support or social participation or trust or emotional support or psychosocial support or community capital or neighbo*rhood cohesion or social influence or (soci*context* or soci*-context*)).mp. | 252891 |
| 9 | Health Status Disparities/ or Health Services Accessibility/ or Health Equity/ or (equit* or health*care disparit* or health care disparit* or health status disparit* or health disparit* or health inequalit* or health inequit* or medically underserved).mp. | 122689 |
| 10 | (potential determinants or "significant correlates of" or (independent correlates or independent association*) or variables associated with or "determinants of" or factors associated with or identif* determinants or (more likely or less likely or just as likely) or "risk factors for" or (significantly related to or significant predictor) or (also adj2 associated with) or (at increased risk or at decreased risk) or association* between or (positively associated or negatively associated) or "differed by" or (were high* amongst or were low* amongst) or (inverse relationship with or inversely associated with or inversely related to) or reverse association or differentially affects or "evidence of a link between" or (significantly adj3 likelihood of) or "protective factors for" or (differ* adj2 according to) or (inverse adj2 gradient) or (positive adj2 gradient) or (negative adj2 gradient) or (trends were adj3 across) or (related to adj3 variable*) or (differences were adj3 explained by) or (significant among or no# significant among)).mp. | 1875894 |
| 11 | 1 or 2 or 3 or 4 or 5 or 6 or 7 or 8 or 9 or 10 | 3330025 |
| 12 | (letter or editorial or historical article).pt. | 1846424 |
| 13 | 11 not 12 | 3187369 |
| 14 | economics/ or exp "costs and cost analysis"/ or economics, dental/ or exp "economics, hospital"/ or economics, medical/ or economics, nursing/ or economics, pharmaceutical/ or (economic$ or cost or costs or costly or costing or price or prices or pricing or pharmacoeconomic$).ti,ab. or (expenditure$ not energy).ti,ab. or (value adj1 money).ti,ab. or budget$.ti,ab. | 882672 |
| 15 | (((energy or oxygen) adj cost) or (metabolic adj cost) or ((energy or oxygen) adj expenditure)).ti,ab. | 27645 |
| 16 | 14 not 15 | 876310 |
| 17 | (letter or editorial or historical article).pt. | 1846424 |
| 18 | 16 not 17 | 841963 |
| 19 | Animals/ not (Animals/ and Humans/) | 4543233 |
| 20 | 18 not 19 | 790283 |
| 21 | Economics.fs. and cost-benefit analysis/ | 71422 |
| 22 | ((Letter or Editorial or Historical article).pt. or Animals/) not humans/ | 4908350 |
| 23 | 21 not 22 | 70280 |
| 24 | 20 and 23 | 65274 |
| 25 | 13 and 24 | 13501 |
| 26 | Health Status Disparities/ or Health Services Accessibility/ or Health Equity/ or (health*care disparit* or health care disparit* or health status disparit* or health disparit* or health inequalit* or health inequit* or medically underserved or health equity or health*care equity or health care equity).mp. | 108045 |
| 27 | 25 and 26 | 1229 |
| 28 | remove duplicates from 27 | **1229** |
