# Supplementary material for: Mapping the evidence on health equity considerations in economic evaluations of health interventions: a scoping review protocol
Source: Syst Rev. 2020 Jan 8;9:6. doi: 10.1186/s13643-019-1257-4 (PMC6950907; doi:10.1186/s13643-019-1257-4)
Supplement: Supplementary file 3 — Additional file 3. Search strategy for grey literature. (docx 16kb) [file 13643_2019_1257_MOESM3_ESM.docx]

**ADDITIONAL FILE 3**

**SEARCH STRATEGY FOR GREY LITERATURE**

**Title: Mapping the evidence on health equity considerations in economic evaluations of health interventions: a scoping review protocol.**

| Dates for Search: | June 11 – June 20, 2019 |
| --- | --- |
| Keywords: | Equity, Disparity, Economic, Cost, Health |
| Limits: | No date or language limits used |

Relevant websites from the following sections of the CADTH grey literature checklist, “Grey matters: a practical tool for searching health-related grey literature” (<https://www.cadth.ca/grey-matters>) were searched:

- Health Economics
  - Canada
    - Hospital for Sick Children (Toronto), Paediatric Economic Database Evaluation (PEDE): 31 records
    - Institute of Health Economics (IHE). Publications: 15 records
    - McMaster University, Centre for Health Economics and Policy Analysis. Publications database (CHEPA): 42 records
    - Toronto Health Economics and Technology Assessment Collaborative (THETA): 0 records
  - International
    - Agency for Healthcare Research and Quality (AHRQ): 51 records
    - Federal Reserve Bank of St. Louis. Economic Research Division (IDEAS database): 174 records
    - International Society for Pharmacoeconomics and Outcomes Research (ISPOR): 9 records
    - National Centre for Pharmacoeconomics (NCPE) Ireland: 0 records
    - NHS Economic Evaluation Database (EED), economic evaluations of health care interventions: 34 records
    - University of Aberdeen. Health Economics Research Unit (HERU): 57 records
